# Optimizing the Power of Genome-Wide Association Studies by Using Publicly Available Reference Samples to Expand the Control Group

**DOI:** 10.1002/gepi.20482

**Published:** 2010-01-20

**Authors:** Joanna J Zhuang, Krina Zondervan, Fredrik Nyberg, Chris Harbron, Ansar Jawaid, Lon R Cardon, Bryan J Barratt, Andrew P Morris

**Affiliations:** Wellcome Trust Centre for Human Genetics, University of OxfordOxford, United Kingdom; Global Epidemiology, AstraZeneca R&DMölndal, Sweden; Institute of Environmental Medicine, Karolinska InstituteStockholm, Sweden; Discovery StatisticsAstraZeneca, Macclesfield, Cheshire, United Kingdom; Research and Development GeneticsAstraZeneca, Macclesfield, Cheshire, United Kingdom; Fred Hutchinson Cancer Research CenterSeattle, Washington

**Keywords:** genome-wide association study, expanded control group, population structure, multidimensional scaling, model selection

## Abstract

Genome-wide association (GWA) studies have proved extremely successful in identifying novel genetic loci contributing effects to complex human diseases. In doing so, they have highlighted the fact that many potential loci of modest effect remain undetected, partly due to the need for samples consisting of many thousands of individuals. Large-scale international initiatives, such as the Wellcome Trust Case Control Consortium, the Genetic Association Information Network, and the database of genetic and phenotypic information, aim to facilitate discovery of modest-effect genes by making genome-wide data publicly available, allowing information to be combined for the purpose of pooled analysis. In principle, disease or control samples from these studies could be used to increase the power of any GWA study via judicious use as “genetically matched controls” for other traits. Here, we present the biological motivation for the problem and the theoretical potential for expanding the control group with publicly available disease or reference samples. We demonstrate that a naïve application of this strategy can greatly inflate the false-positive error rate in the presence of population structure. As a remedy, we make use of genome-wide data and model selection techniques to identify “axes” of genetic variation which are associated with disease. These axes are then included as covariates in association analysis to correct for population structure, which can result in increases in power over standard analysis of genetic information from the samples in the original GWA study. *Genet. Epidemiol*. 34: 319–326, 2010. © 2010 Wiley-Liss, Inc.

## INTRODUCTION

Identifying genetic variants that influence common complex diseases can provide valuable insights into their pathogenesis, prevention, and treatment. With well-defined clinical cohorts and the availability of high-quality and cost-effective genotyping platforms that capture much of human genetic variation [Barrett and Cardon, 2006; The International HapMap Consortium, 2005], the most recent wave of genome-wide association (GWA) studies have been well powered to detect “moderate” genetic effects. The much-publicized Wellcome Trust Case Control Consortium (WTCCC) was established to explore the utility, design, and analysis of GWA studies, aiming to improve our understanding of the aetiological basis of several common diseases, including coronary artery disease (CAD), type 1 and type 2 diabetes (T1D and T2D), and rheumatoid arthritis (RA). The main WTCCC experiment in 2,000 cases of each of seven diseases and 3,000 shared controls from the United Kingdom was powered to detect common variants with allelic odds ratios of the order of 1.5–1.7 [The Wellcome Trust Case Control Consortium, 2007]. The study identified many novel genetic associations, the majority of which have now been replicated in independent samples from the same and/or different populations [Frayling et al., 2007; Parkes et al., 2007; Samani et al., 2007; Todd et al., 2007; Zeggini et al., 2007]. However, these studies have also highlighted the fact that many loci of more modest effect (allelic odds ratios below 1.5) remain undetected.

**Fig. 1:**
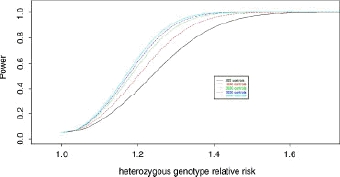
Power of a GWA study of 500 cases to detect association of a causal variant with allele frequency 20% for a range of heterozygous genotype relative risks under a multiplicative model with disease prevalence of 0.1%. Results are presented for a trend test of association for a significance level of 5%, with the number of control samples ranging from 500 to 5,000 individuals.

Statistically, this problem can be regarded as ‘^‘^weak power,^’^’ for which the primary solution is to increase the number of individuals in the study. This is not always technically or financially feasible. However, more and more DNA samples from patients with various diseases, as well as population control cohorts, are being genotyped and subsequently made publicly available through initiatives, such as the WTCCC, the Genetic Association Information Network (GAIN), and the database of genetic and phenotypic information (dbGaP). Thus, there is an opportunity to increase the statistical power of a study at no extra cost, by using the genotype data from these external samples to expand the primary “within-study” control cohort. Figure 1 presents the power of a GWA study of 500 cases to detect association of a causal SNP with risk allele frequency of 20% for a disease of prevalence 0.1%, with the number of control samples ranging from 500 to 5,000 individuals at a significance level of 5%. Power is presented as a function of the heterozygous genotype relative risk under the assumption of a multiplicative disease model. Improvements in power diminish as the ratio of controls to cases increases. When the number of available cases is the limiting factor, a control-case ratio of 4:1 is often cited as optimal, but this is a judgement and the exact rate of diminishing return may vary according to disease risk and allele frequency, as well as analytical issues, such as investigation of interactions or subgroup specific effects. However, if additional genotypes are effectively free, power can be maximized by including as many controls as possible in the study.

Our thoughts on control sample augmentation were initially motivated by the emergence of multisample GWA study designs, such as that employed by the WTCCC, in which there are multiple disease samples drawn from the same, largely homogeneous population [The Wellcome Trust Case Control Consortium, 2007]. Given this internal “genetic matching” and assuming no unmeasured con-founders, the greatest theoretical power to detect genetic effects would be gained by forming an expanded reference group for each disease cohort by combining the primary controls with cases of all other diseases. However, in this type of multisample design, some of the diseases may have overlapping genetic aetiologies, thus inducing genotypic correlations between the different phenotypes due to pleiotropic effects. For example, the main WTCCC experiment includes cases of multiple autoimmune diseases and metabolic disorders. For pleiotropic genes influencing correlated traits, such as *PTPN22* for T1D and RA, making use of cases of one disease as controls for the other can reduce power to detect disease variants, a “dilution” of the genetic effect due to bias in the control group. The inclusion of disease samples in an expanded control group on a genome-wide basis can increase the false-positive error rate, since we may detect associations of large effect with these additional diseases, but may also unexpectedly result in an increase in power for variants influencing traits in opposite directions. However, these issues can be overcome by careful selection of appropriate diseases for expanding the control group and exclusion of SNPs known to be associated with these diseases.

The WTCCC present “expanded reference group analyses,” combining controls with all additional disease samples, increasing the reference group ratio from 1.5:1.0 for each disease to up to approximately 7.5:1.0. Such an analysis is mutually beneficial to all diseases represented in the multisample study. To allow for pleiotropic effects, only nonautoimmune diseases were used in the expanded reference groups for T1D, RA, and Crohn's disease (CD), and CAD and hypertension were not tested against each other. Application of these expanded reference group analyses resulted in more significant evidence of association at the most compelling loci identified in the primary analysis, as well as for established loci for T1D, T2D, and CD.

The challenge we address here is that of expanding the control group to include genotyped individuals from a variety of studies that may not have been ascertained from the same population, and thus may not be genetically matched to the primary “within-study” cases and controls. Inappropriate genetic matching of cases and controls in the presence of population structure can lead to inflation in the false-positive (i.e. type I) error rate, unless properly accounted for in the analysis. A variety of statistical methods exist for the detection of and adjustment for population structure in GWA studies [Devlin and Roeder, 1999; Patterson et al., 2006; Price et al., 2006; Pritchard et al., 2000]. Principal components analysis (PCA) was originally applied to genetic data to infer worldwide axes of human genetic variation from allele frequency differences between populations [Cavalli-Sforza et al., 1993; Menozzi et al., 1978]. The EIGENSTRAT method makes use of axes of genetic variation, estimated from genome-wide genotype data, to continuously adjust the genotypes and phenotypes by amounts attributable to ancestry along each of these axes [Patterson et al., 2006]. By then, testing for association between these ancestry adjusted phenotype and genotype values, cases and controls are effectively genetically matched, thus correcting for the underlying genetic population structure and reducing the inflation in the false-positive error rate [Price et al., 2006].

Here, we make use of a related statistical technique to adjust for population structure with an expanded control cohort. Axes of genetic variation are defined by application of classical multidimensional scaling (MDS) techniques [Cox and Cox, 1994] to a matrix of identity by state (IBS) values between all pairs of samples in the study (cases and the expanded control cohort), using genome-wide genotype data. Such an approach has been used to identify population outliers in the WTCCC [The Wellcome Trust Case Control Consortium, 2007] and for SNP selection and subsequent visualization of population structure [Miclaus et al., 2009]. In a logistic regression framework, we identify which of the resulting axes of genetic variation are associated with disease. These are then treated as covariates in the logistic regression model, providing a basis for testing for association with disease, adjusted for the effects of underlying population structure. We present a simulation study to investigate the false-positive error rate of this procedure, and provide evidence that we can correct for substantial population structure between cases and controls from the original study and the diverse external reference samples used to expand the control cohort, certainly to the extent of allele frequency differences we expect between European populations. We also demonstrate that the use of an expanded control group with adjustment for axes of genetic variation can be more powerful than analysis of the samples from the original GWA study alone.

## METHODS

Consider a population-based sample of cases and controls, which we expect to be ascertained from a genetically homogeneous population, or genetically frequency matched for ethnicity or location in a potentially heterogeneous or admixed population. We assume that we have a series of genetically unmatched external samples that are used to expand the control group in our goal to increase power. All samples are genotyped for the same *N* SNPs, genome-wide. We develop a statistical method to test for association of genetic markers with disease by comparing genotype frequencies between cases and the expanded control cohort that corrects for population structure, when it exists, thus retaining the correct false-positive error rate.

Assuming a multiplicative model of disease risk, we denote the genotype of the *i*th individual at the *k*th SNP by *G_ik_*, coded as 0, 1, or 2, according to the number of minor alleles they carry. We calculate the identity by state (IBS) between each pair of samples, from the original GWA study and all external cohorts, over the whole genome, given by


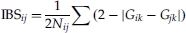


for the *i*th and *j*th samples, where *N_ij_* is the total number of SNPs with genotype data available for both samples. Note that for GWA genotyping platforms, suitably thinned subsets of SNPs should be used to minimize the effects of linkage disequilibrium (LD) between markers (e.g. SNPs with *r*^2^ <0.2 between each other). IBS can take values between 0 and 1, where 1 represents complete identity between a pair of samples. Classical multidimensional scaling is then applied to the matrix of distances, 1-IBS, to identify axes of genetic variation between samples. The resulting scores for the *i*th individual on these axes are denoted as *x*_*i*1_,*x*_*i*2_,…,*x*_*iT*_, where *x_it_* corresponds to the axis with the tth largest eigenvalue.

We begin by testing for association between disease and the first axis of genetic variation. In a logistic regression framework, we model the log-odds of disease of the *i*th individual as


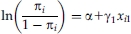


where γ_1_ denotes the effect of the first axis. We perform a likelihood ratio test of association, γ_1_ = 0, and retain the effect in the model if *P*<0.05. We then proceed in a forward selection manner, testing for association of each axis of genetic variation, *x_t_*, in turn, in order of decreasing eigenvalue, retaining any effect in the model for which *P* <0.05. We define an indicator variable *z_t_*, taking the value 1 if the *t*th axis of variation is associated with disease (*P* <0.05) and 0 otherwise. In the absence of population structure, we would expect no axes of genetic variation to be retained in the model, and hence *z_t_* = 0 for all *t*.

Next, we test for association of each SNP, *k*, in turn with disease, adjusting for the effects of population structure by inclusion of the significant axes of genetic variation as covariates in the logistic regression framework. Specifically, we now model the log-odds of disease of the *i*th individual as


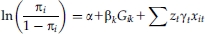


where β_*k*_ is the allelic log-odds ratio of the minor allele at the *k*th SNP, and *z_t_* is defined above. A likelihood ratio test of β_*k*_ = 0 provides a “trend test” of association, adjusted for the effects of population structure. Within this framework, we can account for nongenetic (“environmental”) risk factors as additional covariates in the model and can incorporate alternative coding of the SNP genotypes to allow for different models of genetic risk, such as recessive, dominant, or heterozygote advantage.

## SIMULATION STUDY

We carry out simulations to assess the false-positive error rate and power of the test procedure described above. For each replicate of data, we generate cases and controls from the same source population, together with additional samples from three external cohorts, not necessarily genetically matched for population. These external samples will be used to expand the control cohort, but are not phenotyped for the disease of interest. We specify the divergence between the case-control source population and each external cohort by means of *F_ST_* [Weir, 1996], where *F_ST_* = 0 corresponds to equal allele frequencies at any given SNP across all populations.

For each individual, we simulate genotype data at 10,000 uncorrelated SNPs not associated with disease, but used to calculate IBS relationships. For each SNP, we simulate a minor allele frequency, *q*, in the interval [0.05, 0.5] in the case-control source population, and generate genotypes assuming Hardy-Weinberg equilibrium, irrespective of disease status. We then generate the allele frequency for the same SNP, in each external cohort in turn, using the Balding-Nichols model [Balding and Nichols, 1995]. Specifically, the allele frequency is simulated from a Beta(*a,b*) distribution, where *a = q*(1 − *F_ST_*)/*F_ST_* and *b* = (1−*q*)(1-*F_ST_*)/*F_ST_* [Devlin et al., 2001]. Again, genotypes are simulated with this allele frequency under the assumption of Hardy-Weinberg equilibrium.

Next, we simulate genotypes at the disease SNP in each individual, where the prevalence is fixed at 0.1%. For a fixed high-risk allele frequency and heterozygous genotype relative risk, genotypes are generated for each case-control sample assuming Hardy-Weinberg equilibrium and a multiplicative disease model. In the same way as before, the disease SNP allele frequency is generated in each external cohort using the Balding-Nichols model. Given that the external samples are unselected with respect to the disease phenotype of interest, their genotypes are generated under the assumption of Hardy-Weinberg equilibrium, irrespective of the disease model.

For each replicate of data, we apply classical multidimensional scaling to the IBS relationships between each pair of samples. Given that we simulate four populations (the original source population and three external cohorts), we would expect to require up to three axes of genetic variation to distinguish between them. We thus utilize the forward-selection procedure, described above, to determine which of the first three axes of genetic variation are correlated with phenotype. Three likelihood ratio trend tests of association are then performed:

- T_CC: cases against controls from the source population, without correction for population structure.
- T_F: cases against control cohort expanded by external samples, without correction for population structure.
- T_Fmds: cases against control cohort expanded by external samples, corrected for correlated axes of genetic variation determined through MDS.

For simulations, each replicate of data is limited to 100 cases, 100 controls, and 100 samples from each of the external cohorts. This is to minimize the calculation time for IBS relationships, which is computationally intensive when performed in thousands of replicates. Although we recognize that this sample size is unrealistic for GWA studies, it is sufficient to assess false-positive error rates and compare the relative power of the three association tests.

## RESULTS

### FALSE-POSITIVE ERROR RATE

Table I presents the false-positive error rate of the three trend tests of association over 5,000 replicates of data. Results are presented for a significance level of 5% over a range of *F_ST_*, encompassing no divergence in allele frequencies between the case-control source population and the external cohorts (*F_ST_* = 0), through to the level of differentiation expected between European and African populations. Also presented are mean maximum likelihood estimates of the allelic odds ratio, together with 5- and 95-percentiles across the 5,000 replicates of data.

**Table I:** False-positive error rate (FPER) of three trend tests of association over 5,000 replicates of 100 cases, 100 controls and 100 samples from each of three external cohorts: T_CC, cases against controls from the source population, without correction for population structure; T_F, cases against control cohort expanded by external samples, without correction for population structure; T_Fmds, cases against control cohort expanded by external samples, corrected for up to three axes of genetic variation determined through MDS

	T_CC			T_F			T_Fmds		
			
		Allelic odds ratio			Allelic odds ratio			Allelic odds ratio	
						
*F_ST_*	FPER	Mean	5–95%	FPER	Mean	5–95%	FPER	Mean	5–95%
0	5.0%	1.00	0.66–1.52	5.0%	0.99	0.71–1.36	4.9%	0.99	0.71–1.37
0.001	5.6%	1.00	0.65–1.53	5.4%	0.99	0.70–1.37	5.3%	0.99	0.70–1.38
0.002	5.2%	1.00	0.65–1.54	5.6%	0.99	0.70–1.39	5.6%	0.99	0.70–1.40
0.005	5.1%	0.99	0.64–1.51	6.9%	0.99	0.69–1.40	6.4%	0.99	0.66–1.45
0.01	5.0%	1.00	0.66–1.53	8.3%	1.00	0.69–1.44	6.3%	1.00	0.65–1.53
0.02	5.3%	1.00	0.65–1.53	11.7%	1.00	0.66–1.50	6.1%	0.99	0.64–1.54
0.05	4.9%	1.00	0.66–1.52	21.5%	1.01	0.60–1.68	5.2%	1.00	0.65–1.54
0.1	5.2%	1.00	0.66–1.52	31.6%	1.03	0.56–1.96	5.5%	1.00	0.65–1.54

Mean maximum likelihood estimates of the allelic odds ratio are presented, together with the 5- and 95-percentiles over 5,000 replicates of data. Results are presented for varying degrees of population structure, represented by *F_ST_*, for a significance level of 5%.

As expected, the false-positive error rate of T_CC is correctly maintained at the significance level, regardless of *F_ST_*, since these samples are ascertained from the same population. Expanding the controls with external samples dramatically inflates the false-positive error rate of T_F, as the external cohorts become increasingly divergent from the case-control source population. The test becomes biased in an anticonservative direction for *F_ST_*, even as low as 0.002, which we might expect between populations of Northern and Western European ancestry, for example. For T_CC and T_Fmds, the mean allelic odds ratios are unbiased estimates of the heterozygous genotype relative risk, as expected under a multiplicative disease model, whilst for T_F there is some inflation for the mean extreme models of population structure.

Correcting for population structure in the expanded control group by adjustment for axes of genetic variation controls the false-positive error rate of T_Fmds, remaining consistent with the significance level even for the most extreme population structure. There is some evidence of inflation of the false-positive error rate for moderate levels of population structure. For example, for an *F_ST_* of 0.01, the false-positive error rate is 6.3 ± 0.35% at a significance level of 5%. We hypothesized that with fine-scale population structure, this could be as a result of insufficient SNPs, genome-wide, used to assess the IBS relationships between samples. We thus repeated the simulation using 100,000 uncorrelated SNPs for this calculation, as opposed to 10,000 (supplementary Table I). Based on 5,000 replicates of data, the false-positive error rate dropped to 4.8 ± 0.30% at a significance level of 5%, adequately correcting for the fine scale structure between samples in the expanded control cohort.

### POWER

**Fig. 2:**
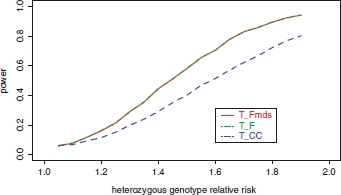
Power of three trend tests of association at a 5% significance level for a high-risk allele frequency of 20% as a function of the allelic odds ratio in the absence of population structure (*F_ST_* = 0): T_CC, cases against controls from the source population, without correction for population structure; T_F, cases against control cohort expanded by external samples, without correction for population structure; T_Fmds, cases against control cohort expanded by external samples, corrected for up to three axes of genetic variation determined through MDS. Power is estimated over 5,000 replicates of 100 cases, 100 controls, and 100 samples from each of three external cohorts.

Figure 2 presents the power of the three trend tests of association at a 5% significance level for a high-risk allele frequency of 20%, as a function of the allelic odds ratio. Power is estimated over 5,000 replicates of data, with no divergence between the case-control population and the external cohorts (*F_ST_* = 0). As expected, both tests that make use of the expanded control cohort are noticeably more powerful than T_CC. However, more importantly, there is no difference in power between T_F and T Fmds. Thus, by adjusting only for those axes of genetic variation that are correlated with phenotype, we take account only of population structure when it exists, and thus do not penalize T_Fmds. The same conclusions are reached, irrespective of high-risk allele frequency (see supplementary Fig. 1 for a high-risk allele frequency of 5%).

**Fig. 3:**
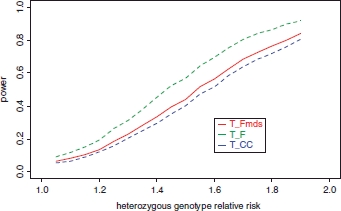
Power of three trend tests of association at a 5% significance level for a high-risk allele frequency of 20%, as a function of the allelic odds ratio in the presence of population structure (*F_ST_* = 0.01): T_CC, cases against controls from the source population, without correction for population structure; T_F, cases against control cohort expanded by external samples, without correction for population structure; T_Fmds, cases against control cohort expanded by external samples, corrected for up to three axes of genetic variation determined through MDS. Power is estimated over 5,000 replicates of 100 cases, 100 controls, and 100 samples from each of three external cohorts.

Figure 3 presents the power of the three trend tests of association at a 5% significance level, this time for external cohorts which are divergent from the case-control source population (*F_ST_* = 0.01), otherwise with the same simulation parameters as before. This time, there is some loss of power for T_Fmds compared to T_F. However, T_F is an anticonservative test of association in this setting (false-positive error rate is 8.3%), and thus is inappropriate. Furthermore, despite the reduction in power of T_Fmds, it remains more powerful than T_CC. The same conclusions are reached, irrespective of high-risk allele frequency (see supplementary Fig. 2 for a high-risk allele frequency of 5%).

Table II presents the power of the three trend tests of association at a 5% significance level for a high-risk allele frequency of 20% and an allelic odds ratio of 1.5, illustrating in more detail the effect of varying levels population structure. Power is estimated over 5,000 replicates of data, for a range of *F_ST_* between the case-control population and external cohorts. Also presented are mean maximum likelihood estimates of the allelic odds ratio, together with 5- and 95-percentiles across the 5,000 replicates of data. Clearly, T_F is most powerful for large *F_ST_*, but it is anticonservative, and thus is not a valid test of association. T_Fmds is, in principle, more powerful than T_CC, although the difference between the tests decreases and approaches zero, as *F_ST_* becomes very large. This is a result of the fact that the external samples are so genetically different from the case-control source population that they add very little information to the disease association after adjustment for the axes of genetic variation.

**Table II:** Power of three trend tests of association for a SNP with minor allele frequency of 20% and a heterozygous genotype relative risk of 1.5 over 5,000 replicates of 100 cases, 100 controls and 100 samples from each of three external cohorts: T_CC, cases against controls from the source population, without correction for population structure; T_F, cases against control cohort expanded by external samples, without correction for population structure; T_Fmds, cases against control cohort expanded by external samples, corrected for up to three axes of genetic variation determined through MDS

	T_CC			T_F			T_Fmds		
			
		Allelic odds ratio			Allelic odds ratio			Allelic odds ratio	
						
*F_ST_*	Power	Mean	5–95%	Power	Mean	5–95%	Power	Mean	5–95%
0	40.3%	1.51	1.03–2.26	59.5%	1.50	1.11–2.02	59.6%	1.51	1.11–2.03
0.001	41.1%	1.51	1.02–2.28	59.7%	1.50	1.10–2.04	59.5%	1.51	1.10–2.05
0.002	40.5%	1.51	1.00–2.30	58.7%	1.50	1.08–2.06	58.6%	1.51	1.08–2.08
0.005	40.5%	1.51	1.03–2.25	57.2%	1.49	1.09–2.08	50.4%	1.51	1.05–2.18
0.01	40.4%	1.51	1.00–2.29	58.2%	1.50	1.05–2.13	45.1%	1.53	1.01–2.33
0.02	41.0%	1.51	1.02–2.26	57.3%	1.50	1.01–2.23	42.7%	1.53	1.02–2.31
0.05	41.1%	1.51	1.03–2.27	57.7%	1.52	0.93–2.48	42.4%	1.53	1.01–2.31
0.1	41.0%	1.52	1.00–2.29	58.0%	1.53	0.82–2.90	41.8%	1.53	1.01–2.31

Mean maximum likelihood estimates of the allelic odds ratio are presented, together with the 5- and 95-percentiles over 5,000 replicates of data. Results are presented for varying degrees of population structure, represented by *F_ST_*, for a significance level of 5%.

Our previous simulations (Table I and supplementary Table I) suggest that application of MDS to IBS metrics calculated from 100,000 uncorrelated SNPs can distinguish fine-scale population structure, which is not apparent when only 10,000 SNPs are used. Supplementary Table II presents the power of the three trend tests of association at a 5% significance level for a high-risk allele frequency of 20% and an allelic odds ratio of 1.5, this time using 100,000 uncorrelated SNPs in calculating the IBS matrix. Comparing these results to those in Table II, we clearly demonstrate a noticeable drop in power to detect association for *F_ST_* <0.01. With more SNPs, we are better able to distinguish between samples from even relatively closely related populations, with the result that external samples provide little additional information, even at modest levels of population structure. This issue is common to many methods for detecting population structure: a trade-off between sensitivity to fine-scale stratification and correction for its effects in subsequent association analysis.

### SENSITIVITY TO SELECTION STRATEGY FOR INCLUSION OF AXES OF GENETIC VARIATION IN ADJUSTED ANALYSES

Throughout the simulations presented above, we have adjusted only for those axes of genetic variation amongst the first three, which are correlated with disease status using *P* <0.05. For example, if only the first and third of these axes are correlated with disease status, we do not make adjustment for the second. We have investigated the sensitivity of our approach to this selection strategy by repeating simulations: (i) adjusting only for those axes of genetic variation amongst the first three that are correlated with disease status using *P* <0.1 and (ii) all of the first three axes of genetic variation, irrespective of their correlation with disease status.

Supplementary Table III presents the false-positive error rate of the trend test for association, T_Fmds, adjusting for up to three axes of genetic variation, according to the three selection criteria described above. Results are presented over 5,000 replicates for a range of *F_ST_*, for a significance level of 5%. The selection criteria makes little difference to the false-positive error rate, presumably because the inclusion of axes of genetic variation in the logistic regression model that do not correlate with disease does not impact on the association analysis, because the additional degrees of freedom required are small compared to the sample size. A similar conclusion is reached in terms of the effect of selection criteria on power, as presented in supplementary Table IV, for a SNP with minor allele frequency of 20% in the source population and a heterozygous genotype relative risk of 1.5. However, if we were to include many more axes of genetic variation without selection, we would expect larger differences in the false-positive error rate and power between approaches, particularly in the absence of population structure. Therefore, one clear advantage of the selection approach is that we can test large numbers of axes of genetic variation and include only those relevant to disease association, thus minimizing any loss in power.

To address this assertion, we have also investigated the sensitivity of our approach to the number of axes of genetic variation, by adjusting only for those axes of genetic variation, this time amongst the first ten, which are correlated with disease status using *P* <0.05. Supplementary Table V presents the false-positive error rate of the trend test for association, T_Fmds, adjusting for up to three or up to ten axes of genetic variation. Results are presented over 5,000 replicates for a range of *F_ST_*, for a significance level of 5%. Also presented is the proportion of replicates of simulated data in which more than the first three axes of genetic variation were selected to adjust for population structure. The choice of the number of axes of genetic variation used for adjustment has little impact on the false-positive error rate, with less than 10% of replicates of data utilizing more than the first three axes. There is also little effect on power, demonstrated in supplementary Table VI for a SNP with minor allele frequency of 20% frequency in the source population, and a heterozygous genotype relative risk of 1.5.

## DISCUSSION

The increasing availability of genome-wide genotype data from public databases of individuals from a wide range of disease cohorts offers an exciting opportunity to increase sample sizes utilized in GWA studies. Provided that samples are from the same population and assuming no unmeasured confounders, power can be increased by expanding the control cohort with disease samples from other studies. However, in the presence of unobserved population structure, such an approach can lead to an increase in the false-positive error rate. Here, we demonstrate by simulation that correction for axes of genetic variation from MDS, obtained from IBS metrics calculated from large numbers of SNPs, genome-wide maintains the correct false-positive error rate, even in the presence of genetic differences between samples in the expanded control group of the magnitude we might expect across Europe or even further afield. In the absence of population structure, there is no loss in power compared to a test that does not correct for axes of genetic variation. Furthermore, there is a substantial increase in power over studies that do not make use of external reference samples as controls. This approach has broad applicability to GWA studies, making use of the increasing number of raw genotype data sets which are being made publicly available to increase the power to detect novel disease loci, whilst remaining computationally tractable and rigorous to population structure.

There are a number of potential problems in the application of our approach to expanding the control cohort. First, there may be overlapping aetiologies between cases and the disease cohorts used to expand the control cohort, for example, cases of T1D and additional samples from other autoimmune disease cohorts. Thus, by including diseases of overlapping aetiologies in the case and control cohorts, we reduce our power to detect association with pleiotropic genes. A second problem arises as a result of strong signals of association among cohorts utilized to expand the control group. False-positive signals of association for the disease of interest will be identified unless sufficiently large numbers of samples from other reference cohorts are included as controls. Both of these problems highlight the need for careful selection of cohorts used to expand the control group and care in the interpretation of the results of such analyses. In addition, increases in the false-positive error rates of association tests applied to expanded control cohorts can arise as a result of systematic genotyping error differences between studies. It is crucial, therefore, to follow up any positive signals of association with genotyping in independent samples for replication.

Interpretation of the axes of genetic variation is crucial before testing their association with disease. In a homogeneous population, the first components of genetic variation may reflect local extended LD patterns rather than genome-wide structure, such as that reported in the main WTCCC experiment. Clearly, if the region of extended LD contains causal variants, regressing out the corresponding axis of genetic variation will prevent their detection. This is likely to be true for autoimmune diseases in the MHC, for example, an extended region of strong LD harboring many established associations. Apparent axes of genetic variation may also reflect “batch effects,” resulting from the use of different genotyping platforms or calling algorithms between cohorts, which will be important to take account of in the same way as population structure, in order to reduce the inflation in false-positive error rates.

The approach outlined here could be utilized to expand the case cohort with additional samples of the same disease, even if not directly matched for population, in the same way as controls. However, if gene-environment interaction is likely to exist for the disease under investigation, it is also important to match cases for exposure to potential environmental risk factors, or at least to measure these variables, which may not be possible with publicly available samples. Furthermore, there may be allelic- or genetic-heterogeneity between populations, particularly for complex traits. Under such circumstances, combining case cohorts would reduce power to detect association, and thus would not be advised without prior evidence to the contrary.

Many GWA genotyping platforms are available, meaning that publicly available samples may not always have been genotyped for the same set of SNPs. However, imputation techniques [Marchini et al., 2007] allow us to combine samples, irrespective of genotyping platform, by making use of reference samples of haplotypes, such as those in the International HapMap project [The International HapMap Consortium, 2005], and simple models of population genetics to approximate the distribution of genotype cells carried by individuals not directly genotyped on the GWA array. In this way, samples that are directly genotyped for one SNP can be combined with samples for which the same SNP has been imputed, again leading to an increase in power.
