# Supplementary material for: Optimizing the Power of Genome-Wide Association Studies by Using Publicly Available Reference Samples to Expand the Control Group
Source: Genet Epidemiol. 2010 Jan 20;34(4):319–26. doi: 10.1002/gepi.20482 (PMC2962805; doi:10.1002/gepi.20482)

Supplementary Table I. False positive error rate (FPER) of three trend tests of association over 5,000 replicates of 100 cases, 100 controls and 100 samples from each of three external cohorts: T_CC, cases against controls from the source population, without correction for population structure; T_F, cases against control cohort expanded by external samples, without correction for population structure; T_Fmds, cases against control cohort expanded by external samples, corrected for up to three axes of genetic variation determined through MDS using 100,000 uncorrelated SNPs. Mean maximum likelihood estimates of the allelic odds ratio are presented, together with the 5- and 95-percentiles over 5,000 replicates of data. Results are presented for varying degrees of population structure, represented by F_{ST}, for a significance level of 5%.

F_{ST}	T_CC			T_F			T_Fmds		
	FPER	Allelic odds ratio		FPER	Allelic odds ratio		FPER	Allelic odds ratio	
		Mean	5-95%		Mean	5-95%		Mean	5-95%
0	5.4%	1.00	0.65-1.52	5.5%	0.99	0.70-1.37	5.5%	0.99	0.70-1.37
0.001	4.9%	1.00	0.65-1.51	5.5%	0.99	0.70-1.38	5.6%	0.99	0.70-1.40
0.002	4.6%	1.00	0.66-1.52	6.2%	0.99	0.70-1.39	5.7%	1.00	0.65-1.49
0.005	5.4%	1.00	0.65-1.52	6.6%	0.99	0.70-1.40	5.9%	0.99	0.64-1.54
0.01	4.6%	1.00	0.66-1.51	8.6%	1.00	0.68-1.45	4.8%	1.00	0.66-1.51
0.02	5.0%	1.00	0.66-1.53	11.6%	1.00	0.67-1.49	5.2%	1.00	0.65-1.55
0.05	5.5%	1.00	0.64-1.52	22.5%	1.01	0.60-1.69	5.6%	1.00	0.64-1.53
0.1	5.2%	1.00	0.66-1.52	32.0%	1.03	0.56-1.98	5.4%	1.00	0.66-1.52

Supplementary Table II. Power of three trend tests of association for a SNP with minor allele frequency of 20% and a heterozygous genotype relative risk of 1.5 over 5,000 replicates of 100 cases, 100 controls and 100 samples from each of three external cohorts: T_CC, cases against controls from the source population, without correction for population structure; T_F, cases against control cohort expanded by external samples, without correction for population structure; T_Fmds, cases against control cohort expanded by external samples, corrected for up to three axes of genetic variation determined through MDS using 100,000 uncorrelated SNPs. Mean maximum likelihood estimates of the allelic odds ratio are presented, together with the 5- and 95-percentiles over 5,000 replicates of data. Results are presented for varying degrees of population structure, represented by F_{ST}, for a significance level of 5%.

F_{ST}	T_CC			T_F			T_Fmds		
	Power	Allelic odds ratio		Power	Allelic odds ratio		Power	Allelic odds ratio	
		Mean	5-95%		Mean	5-95%		Mean	5-95%
0	40.7%	1.51	1.03-2.29	59.0%	1.50	1.10-2.04	58.9%	1.50	1.10-2.04
0.001	41.0%	1.52	1.03-2.28	59.2%	1.50	1.10-2.03	57.7%	1.50	1.09-2.05
0.002	39.7%	1.51	1.03-2.27	58.6%	1.50	1.10-2.06	46.7%	1.52	1.04-2.22
0.005	41.0%	1.52	1.03-2.30	57.6%	1.50	1.08-2.07	43.6%	1.53	1.03-2.31
0.01	41.0%	1.52	1.03-2.29	58.8%	1.50	1.06-2.12	41.9%	1.53	1.03-2.31
0.02	40.0%	1.51	1.00-2.27	57.1%	1.50	1.01-2.24	40.7%	1.51	1.00-2.29
0.05	41.1%	1.51	1.03-2.25	58.6%	1.52	0.94-2.56	41.5%	1.51	1.02-2.25
0.1	40.1%	1.51	1.00-2.27	58.9%	1.53	0.82-2.91	40.6%	1.51	1.01-2.26

Supplementary Table III. False positive error rate (FPER) of the trend tests of association, adjusting for up to three axes of genetic variation (T_Fmds), over 5,000 replicates of 100 cases, 100 controls and 100 samples from each of three external cohorts. Mean maximum likelihood estimates of the allelic odds ratio are presented, together with the 5- and 95-percentiles over 5,000 replicates of data. Results are presented for a range of F_{ST} for a significance level of 5%: (a) adjusting for each of the first three axes of genetic variation that are correlated with disease status using $p<0.05$; (b) adjusting for each of the first three axes of genetic variation that are correlated with disease status using $p<0.1$; and (c) all of the first three axes of genetic variation, irrespective of their correlation with disease status.

F_{ST}	Axes with $p<0.05$			Axes with $p<0.1$			All axes		
	FPER	Allelic odds ratio		FPER	Allelic odds ratio		FPER	Allelic odds ratio	
		Mean	5-95%		Mean	5-95%		Mean	5-95%
0	4.9%	0.99	0.71-1.37	4.6%	0.99	0.71-1.36	5.4%	1.00	0.71-1.39
0.001	5.3%	0.99	0.70-1.38	5.4%	1.00	0.71-1.38	5.4%	1.00	0.71-1.39
0.002	5.6%	0.99	0.70-1.40	5.8%	0.99	0.70-1.38	5.4%	1.00	0.70-1.39
0.005	6.4%	0.99	0.66-1.45	6.2%	1.00	0.67-1.47	6.4%	1.00	0.67-1.46
0.01	6.3%	1.00	0.65-1.53	5.9%	1.00	0.65-1.53	6.5%	1.00	0.64-1.55
0.02	6.1%	0.99	0.64-1.54	5.5%	1.00	0.64-1.53	6.4%	1.00	0.64-1.55
0.05	5.2%	1.00	0.65-1.54	5.5%	1.00	0.65-1.54	5.4%	1.00	0.65-1.53
0.1	5.5%	1.00	0.65-1.54	5.5%	1.00	0.65-1.53	5.2%	1.01	0.66-1.54

Supplementary Table IV. Power of the trend test of association, adjusting for up to three axes of genetic variation (T_Fmds), for a SNP with minor allele frequency of 20% and a heterozygous genotype relative risk of 1.5 over 5,000 replicates of 100 cases, 100 controls and 100 samples from each of three external cohorts. Mean maximum likelihood estimates of the allelic odds ratio are presented, together with the 5- and 95-percentiles over 5,000 replicates of data. Results are presented for a range of F_{ST} for a significance level of 5%: (a) adjusting for each of the first three axes of genetic variation that are correlated with disease status using $p<0.05$; (b) adjusting for each of the first three axes of genetic variation that are correlated with disease status using $p<0.1$; and (c) all of the first three axes of genetic variation, irrespective of their correlation with disease status.

F_{ST}	Axes with $p<0.05$			Axes with $p<0.1$			All axes		
	Power	Allelic odds ratio		Power	Allelic odds ratio		Power	Allelic odds ratio	
		Mean	5-95%		Mean	5-95%		Mean	5-95%
0	59.6%	1.51	1.11-2.03	59.4%	1.50	1.09-2.01	58.8%	1.50	1.09-2.03
0.001	59.5%	1.51	1.10-2.05	58.5%	1.50	1.10-2.05	58.1%	1.50	1.09-2.04
0.002	58.6%	1.51	1.08-2.08	59.3%	1.50	1.08-2.06	59.1%	1.51	1.08-2.08
0.005	50.4%	1.51	1.05-2.18	51.3%	1.52	1.05-2.18	50.1%	1.51	1.05-2.18
0.01	45.1%	1.53	1.01-2.33	46.1%	1.53	1.04-2.27	45.4%	1.53	1.03-2.29
0.02	42.7%	1.53	1.02-2.31	43.5%	1.53	1.02-2.31	43.7%	1.53	1.01-2.32
0.05	42.4%	1.53	1.01-2.31	40.8%	1.52	1.00-2.32	42.0%	1.52	1.03-2.28
0.1	41.8%	1.53	1.01-2.31	40.4%	1.51	1.02-2.30	40.6%	1.52	1.01-2.30

Supplementary Table V. False positive error rate (FPER) of the trend test of association, adjusting for axes of genetic variation (T_Fmds), over 5,000 replicates of 100 cases, 100 controls and 100 samples from each of three external cohorts. Mean maximum likelihood estimates of the allelic odds ratio are presented, together with the 5- and 95-percentiles over 5,000 replicates of data. Results are presented for a range of F_{ST} for a significance level of 5%: (a) adjusting for each of the first three axes of genetic variation that are correlated with disease status using $p<0.05$; (b) adjusting for each of the first then axes of genetic variation that are correlated with disease status using $p<0.05$. Also presented is the proportion of replicates for which more than the first three axes of genetic variation were selected to adjust for population structure.

F_{ST}	Up to three axes			Up to ten axes			
	FPER	Allelic odds ratio		FPER	Allelic odds ratio		>3 axes
		Mean	5-95%		Mean	5-95%	
0	4.9%	0.99	0.71-1.37	5.0%	0.99	0.71-1.38	0.0008
0.001	5.3%	0.99	0.70-1.38	4.7%	1.00	0.71-1.38	0.0094
0.002	5.6%	0.99	0.70-1.40	6.2%	1.00	0.70-1.40	0.0868
0.005	6.4%	0.99	0.66-1.45	5.7%	0.99	0.67-1.45	0.1176
0.01	6.3%	1.00	0.65-1.53	6.2%	0.99	0.64-1.52	0.0118
0.02	6.1%	0.99	0.64-1.54	6.6%	0.99	0.63-1.54	0.0070
0.05	5.2%	1.00	0.65-1.54	6.2%	1.00	0.65-1.55	0.0034
0.1	5.5%	1.00	0.65-1.54	5.1%	1.00	0.66-1.53	0.0048

Supplementary Table VI. Power of the trend test of association, adjusting for axes of genetic variation (T_Fmds), for a SNP with minor allele frequency of 20% and a heterozygous genotype relative risk of 1.5 over 5,000 replicates of 100 cases, 100 controls and 100 samples from each of three external cohorts. Mean maximum likelihood estimates of the allelic odds ratio are presented, together with the 5- and 95-percentiles over 5,000 replicates of data. Results are presented for a range of F_{ST} for a significance level of 5%: (a) adjusting for each of the first three axes of genetic variation that are correlated with disease status using $p<0.05$; (b) adjusting for each of the first then axes of genetic variation that are correlated with disease status using $p<0.05$. Also presented is the proportion of replicates for which more than the first three axes of genetic variation were selected to adjust for population structure.

F_{ST}	Up to three axes			Up to ten axes			
	Power	Allelic odds ratio		Power	Allelic odds ratio		>3 axes
		Mean	5-95%		Mean	5-95%	
0	59.6%	1.51	1.11-2.03	59.1%	1.50	1.10-2.04	0.0014
0.001	59.5%	1.51	1.10-2.05	56.7%	1.49	1.09-2.06	0.0126
0.002	58.6%	1.51	1.08-2.08	57.5%	1.50	1.08-2.06	0.0890
0.005	50.4%	1.51	1.05-2.18	49.1%	1.51	1.04-2.18	0.1120
0.01	45.1%	1.53	1.01-2.33	45.1%	1.53	1.02-2.28	0.0094
0.02	42.7%	1.53	1.02-2.31	41.8%	1.52	1.01-2.32	0.0074
0.05	42.4%	1.53	1.01-2.31	40.9%	1.52	1.00-2.34	0.0048
0.1	41.8%	1.53	1.01-2.31	41.1%	1.52	1.01-2.32	0.0054

List of supplementary figures

Supplementary Figure 1. Power of three trend tests of association at a 5% significance level for a high risk allele frequency of 5% as a function of the allelic odds ratio in the absence of population structure (F_{ST} = 0): T_CC, cases against controls from the source population, without correction for population structure; T_F, cases against control cohort expanded by external samples, without correction for population structure; T_Fmds, cases against control cohort expanded by external samples, corrected for up to three axes of genetic variation determined through MDS. Power is estimated over 5,000 replicates of 100 cases, 100 controls and 100 samples from each of three external cohorts.

Supplementary Figure 2. Power of three trend tests of association at a 5% significance level for a high risk allele frequency of 5% as a function of the allelic odds ratio in the presence of population structure (F_{ST} = 0.01): T_CC, cases against controls from the source population, without correction for population structure; T_F, cases against control cohort expanded by external samples, without correction for population structure; T_Fmds, cases against control cohort expanded by external samples, corrected for up to three axes of genetic variation determined through MDS. Power is estimated over 5,000 replicates of 100 cases, 100 controls and 100 samples from each of three external cohorts.

Supplementary Figure 1.

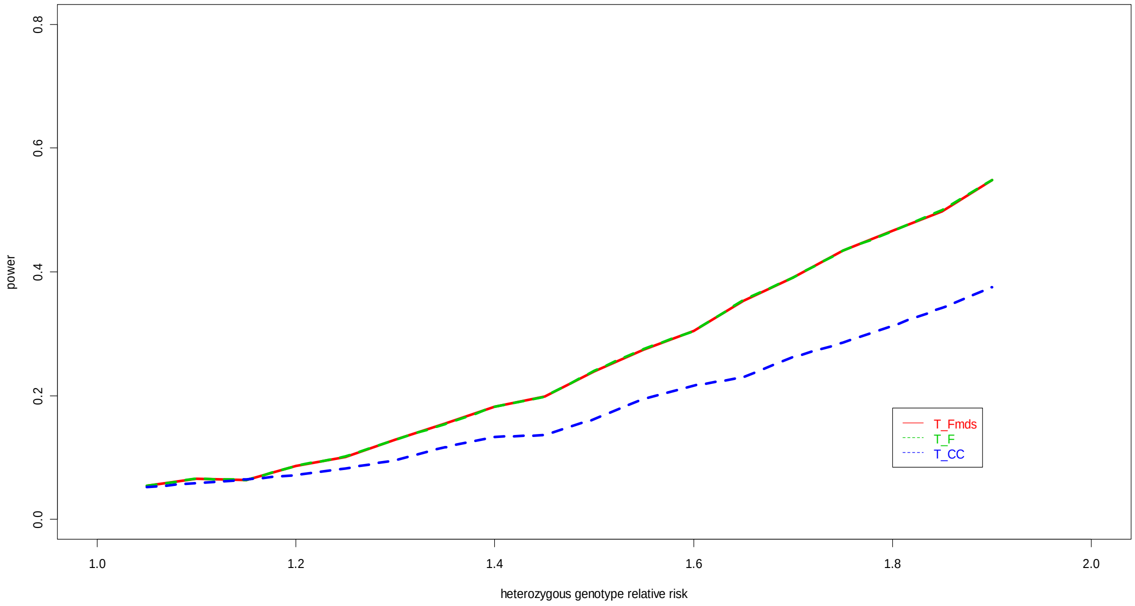

Supplementary Figure 2.

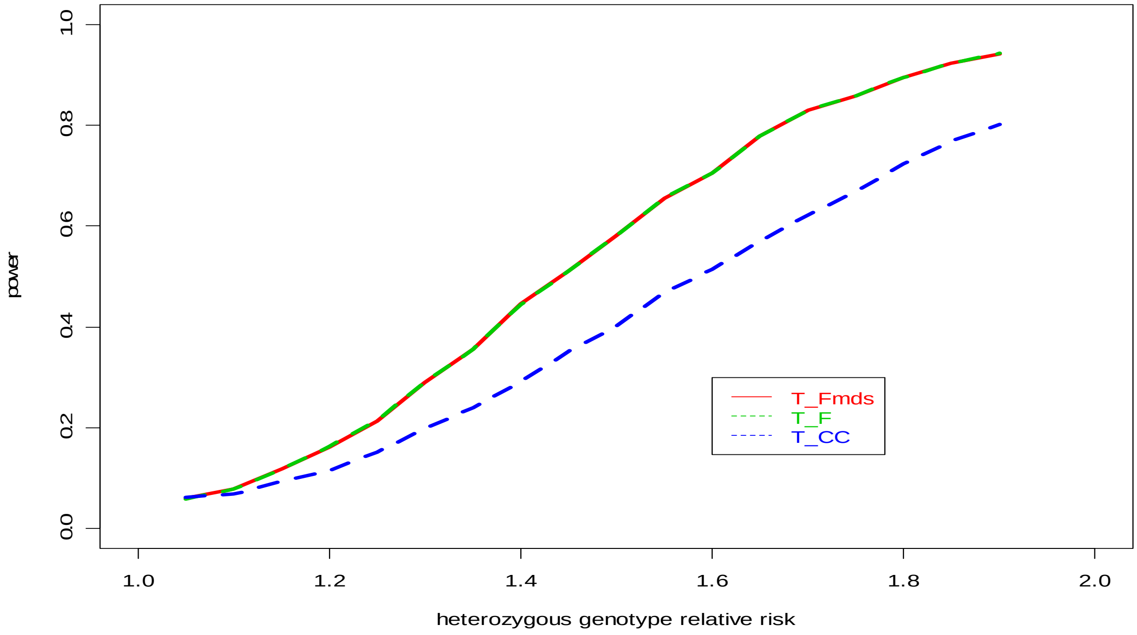

Supplement: Supplementary file 1 [file gepi0034-0319-SD1.pdf]
